# *hOGG1* Ser326Cys Polymorphism and Risk of Hepatocellular Carcinoma among Japanese

**DOI:** 10.2188/jea.16.233

**Published:** 2006-11-03

**Authors:** Tatsuhiko Sakamoto, Yasuki Higaki, Megumi Hara, Masayoshi Ichiba, Mikako Horita, Toshihiko Mizuta, Yuichiro Eguchi, Tsutomu Yasutake, Iwata Ozaki, Kyosuke Yamamoto, Shingo Onohara, Seiji Kawazoe, Hirohisa Shigematsu, Shunzo Koizumi, Keitaro Tanaka

**Affiliations:** 1 Department of Preventive Medicine, Faculty of Medicine, Saga University.; 2 Department of Social and Environmental Medicine, Faculty of Medicine, Saga University.; 3 Department of Internal Medicine, Faculty of Medicine, Saga University.; 4 Department of Internal Medicine, Saga Prefectural Hospital Koseikan.; 5 Department of General Medicine, Faculty of Medicine, Saga University.

**Keywords:** Carcinoma, Hepatocellular, human 8-oxoguanine glycosylase 1, Polymorphism (Genetics)

## Abstract

**BACKGROUND:**

The Ser326Cys polymorphism in human oxoguanine glycosylase 1 (hOGG1), which is involved in the repair of 8-hydroxy-2-deoxyguanine in oxidatively damaged DNA, has been associated with susceptibility to certain cancers, but has not been examined in causation of hepatocellular carcinoma (HCC).

**METHODS:**

We conducted a case-control study to investigate whether this polymorphism was related to HCC risk with any interaction with alcohol consumption and cigarette smoking. Genotyping was performed by a polymerase chain reaction with confronting two-pair primers among 209 newly diagnosed HCC cases, 275 hospital controls, and 381 patients with chronic liver disease (CLD) without HCC.

**RESULTS:**

Overall, the *hOGG1* genotype was not significantly associated with HCC; adjusted odds ratios (and 95% confidence intervals) for the Ser/Cys and Cys/Cys genotypes compared with the Ser/Ser genotype were 0.79 (0.35-1.79) and 0.48 (0.18-1.27) against hospital controls, and 1.51 (0.96-3.37) and 0.86 (0.50-1.47) against CLD patients. We could not detect any significant gene-alcohol interaction (p = 0.95 or 0.16) or gene-smoking interaction (p = 0.70 or 0.69).

**CONCLUTIONS:**

These results suggest that the *hOGG1* Ser326Cys polymorphism may not play a major role as an independent factor in hepatocarcinogenesis.

The major causative factor of hepatocellular carcinoma (HCC) is chronic infection with hepatitis C virus (HCV) and hepatitis B virus (HBV) in Japan.^1^^,^^2^ Alcohol intake and cigarette smoking have also been implicated in the etiology of HCC.^3^^,^^4^ Although the biological mechanisms underlying these factors are not fully understood, one of the proposed mechanisms represents the involvement of oxidative DNA damage which can induce mutations leading to cancer.^5^^,^^6^ Chronic hepatic inflammation caused by hepatitis viruses and exposure to alcohol and tobacco stimulate the generation of hepatic reactive oxygen species (ROS) causing oxidative DNA damage.^7^^-^^9^

Among many types of oxidative DNA damage, 8-hydroxy-2-deoxyguanine (8-OHdG) is highly mutagenic because of its propensities to mispair with adenine during DNA replication and to cause ultimately GC to TA transversion.^10^^,^^11^ The human 8-oxoguanine glycosylase 1 (hOGG1) encoded by the *hOGG1* gene located on chromosome 3p25/26 has an activity to remove directly 8-OHdG from DNA as a part of the base excision repair pathway.^12^^,^^13^ The Ser326Cys polymorphism in exon 7 of *hOGG1* has been related to glycosylase function and an individual’s ability to repair damaged DNA.^14^^,^^15^

Although recent studies^16^^-^^20^ suggested that the low active *hOGG1* allele (326Cys) was positively associated with the risk of several cancers while showing interactions with environmental factors, the association between this polymorphism and HCC has not been examined so far. Therefore, we conducted this case-control study including 209 HCC cases and two different controls (275 hospital controls and 381 patients with chronic liver disease [CLD] without HCC); CLD patients were selected as control subjects because most HCC patients in Japan have preexisting CLD.

## METHODS

### Subjects

The details of this study have been described elsewhere.^21^ Briefly, all study subjects were restricted to residents of Saga Prefecture, Japan, who were aged 40 to 79 years. Incident HCC cases (n = 209, participation rate = 92%), who were admitted or outpatients of 2 main hospitals in Saga City (Saga Medical School Hospital and Saga Prefectural Hospital) between April 2001 and March 2004, were recruited as case subjects; 198 cases (95%) had preexisting cirrhosis (n = 167) or chronic hepatitis (n = 31). Hospital controls (n = 275, participation rate = 73%) were first-time visitors at the general outpatient clinic of Saga Medical School Hospital between May 2001 and April 2003; these controls were selected so that the sex and age distribution of them would be similar to that of deaths from liver cancer in Saga Prefecture in 1998.21 They had various diseases (n = 190), undiagnosed symptoms (n = 49), or no definite abnormality (n = 36). Patients with CLD (298 patients with chronic hepatitis and 83 patients with cirrhosis, participation rate = 96%) were out- or inpatients of the hospitals same as HCC cases between September 2001 and March 2004; patients with special types of CLD (primary and secondary biliary cirrhosis, autoimmune hepatitis, and liver disease due to parasitotis, congestive heart failure, or metabolic disorders) were excluded. All control subjects had no evidence of HCC.

The study protocol was approved by the ethics committees of the above two hospitals, and written informed consent to the use of their blood and clinical information for this study was obtained from all subjects.

### Interviews

Research nurses interviewed study subjects on alcohol drinking and smoking habits using a uniform questionnaire. A history of heavy drinking was defined as having imbibed 69 g or more of ethanol per day for 10 or more years. We regarded “never smokers” as individuals who had never smoked or had smoked for less than 1 year, “former smokers” as those who stopped smoking 1 or more years before the interview, and “current smokers” as those who currently smoked or stopped smoking less than 1 year prior to the interview. The cumulative amount of smoking was calculated as pack-years.

### Serologic Tests and Genotyping

Venous blood was drawn, and plasma samples were tested for hepatitis B surface antigen (HBsAg) by a chemiluminescent immunoassay (CLIA; Dainabot, Tokyo, Japan) and for antibodies to HCV (HCVAb) by a 2nd-generation enzyme immunoassay (Abott HCV EIA II; Dainabot, Tokyo).

**Figure 1.**
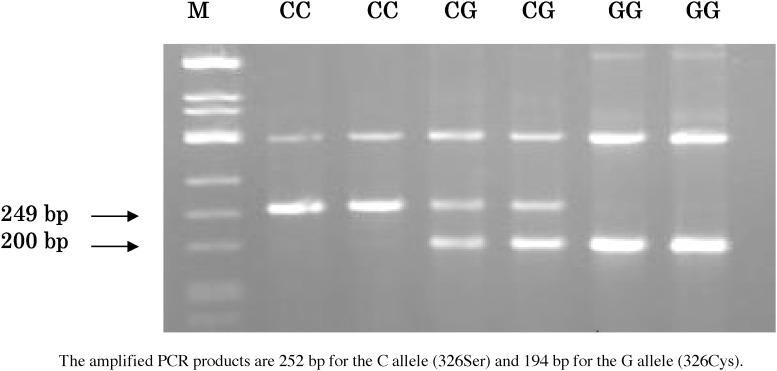
PCR-CTPP analysis for the hOGG1 polymorphism at codon 326 in exon 7.

DNA was extracted from buffy coat preparations by using a commercial kit (QIAmp DNA Blood Mini kit; QIAGEN Inc, Tokyo). The *hOGG1* Ser326Cys polymorphism was genotyped by a polymerase chain reaction (PCR) with confronting two-pair primers (PCR-CTPP) according to Ito et al^22^ Genomic DNA (10-150 ng) was amplified in a volume of 25 *μ*L with 0.18mM dNTPs, 12.5 pmol of each primer, 0.5 units of AmpliTaq Gold (Perkin-Elmer Corp., Foster City, CA), and 2.5 *μ*L of 10×PCR buffer including 15 mM MgCl_2_. The following 4 primers were used for each reaction: F1 (5′-CAGCCCAGACCCAGTGGACTC-3′), R1 (5′-TGGCTCCTGAGCATGGCGGG-3′), F2 (5′-CAGTGCCGACCTGCGCCAATG-3′), and R2 (5′-GGTAGTCACAGGGAGGCCCC-3′). PCR was conducted as follows: a 10 minute initial denature at 95°C, 30 cycles for 1 minute at 95°C, 1 minute at 64°C, and 1 minute at 72°C, and a 5 minute final extension at 72°C. PCR products were subjected to electrophoresis in 2% agarose gels and were visualized with ethidium bromide staining. The primer pair F1 and R1 produced the C allele (Ser326) band (252 bp), while F2 and R2 produced the G allele (326Cys) band (194 bp) (Figure 1). To validate the results, 10% were randomly selected for genotyping by using a PCR-restriction fragment length polymorphism analysis,^16^ and the results were 100% concordant.

### Statistical Analysis

Chi square tests were used for unadjusted comparisons based on frequency. The Wilcoxon’s rank sum test was conducted to compare the distribution of age. Unconditional logistic regression models were used to estimate crude and adjusted odds ratios (ORs) of HCC and their 95 percent confidence intervals (CIs) for *hOGG1* genotypes by using dummy variables, with adjustment for potential confounders including sex, age category (40-49, 50-59, 60-69, and 70-79 years), heavy drinking history, smoking status (never, former, and current smokers), and HBsAg and HCVAb status. The gene-environment interaction on HCC risk was evaluated by including in the model the product terms of variables of interest (i.e., *hOGG1* genotype and heavy drinking history/smoking status), as well as main effects and covariates. Likelihood ratio tests were used to examine the overall statistical significance of a set of interaction terms. Tests of linear trend for *hOGG1* genotypes were performed by assigning an ordinal variable to the genotypes in the logistic model. All statistical analyses were performed with the SAS^®^/PC statistical package (SAS Institute Inc., Cary, NC).

## RESULTS

Selected characteristics of study subjects are shown in Table 1. As compared with hospital controls, HCC cases showed significantly higher prevalences of older subjects (p < 0.01), HBsAg positives (p < 0.01), HCVAb positives (p < 0.01), males with heavy drinking history (p < 0.01), and male current smokers (p = 0.03). As compared to CLD patients, HCC cases revealed significantly greater proportions of males (p < 0.01), older subjects (p < 0.01), and males with heavy drinking history (p < 0.01).

**Table 1.** Selected characteristics of study subjects.

Factor	HCC cases			Hospital controls	CLD patients	P*	P^†^
	n(%)			n(%)	n(%)		
Sex							
Male	141 (67.5)			180 (65.5)	205 (53.8)		
Female	68 (32.5)			95 (34.5)	176 (46.2)	0.64	<0.01

Age (year)							
40-49	6 (2.9)			42 (15.3)	73 (19.2)		
50-59	28 (13.4)			85 (30.9)	93 (24.4)		
60-69	76 (36.4)			86 (31.3)	136 (35.7)		
70-79	99 (47.4)			62 (22.6)	79 (9.2)	<0.01	<0.01
Median	69 yr			61 yr	61 yr	<0.01	<0.01

HBsAg							
Negative	190 (90.9)			269 (97.8)	346 (90.8)		
Positive	19 (9.1)			6 (2.2)	35 (9.2)	<0.01	0.97

HCVAb							
Negative	30 (14.4)			254 (92.4)	54 (14.2)		
Positive	179 (85.7)			21 (7.6)	327 (85.8)	<0.01	0.95

Heavy drinking history (male)							
No	95 (67.4)			158 (87.8)	170 (82.9)		
Yes	46 (32.6)			22 (12.2)	35 (17.1)	<0.01	<0.01

Heavy drinking history (female)							
No	65 (95.6)			94 (99.0)	172 (97.7)		
Yes	3 (4.4)			1 (1.1)	4 (2.3)	0.17	0.37

Smoking status (male)							
Never	24 (17.0)			50 (27.8)	54 (26.3)		
Former	51 (36.2)			67 (37.2)	76 (37.1)		
Current	66 (46.8)			63 (35.0)	75 (36.7)	0.03	0.07

Smoking status (female)							
Never	61 (89.7)			88 (92.6)	150 (85.2)		
Former	4 (5.9)			3 (3.2)	15 (8.5)		
Current	3 (4.4)			4 (4.2)	11 (6.3)	0.70	0.66

*hOGG1* genotype							
Ser/Ser	56 (26.8)			73 (26.5)	105 (27.6)		
Ser/Cys	110 (52.6)			123 (44.7)	176 (46.2)		
Cys/Cys	43 (20.6)			79 (28.7)	100 (26.2)	0.10	0.23

* : Comparisons were made between HCC cases and hospital controls.

† : Comparisons were made between HCC cases and CLD patients.

HCC: hepatocellular carcinoma

CLD: chronic liver diseases

The frequency of *hOGG1* genotype showed no significant difference between HCC cases and either control group (p = 0.10 or 0.23) (Table 1). The genotype distributions for hospital controls and CLD patients were in Hardy-Weinberg equilibrium (p = 0.08 and 0.14, respectively). After adjustment for sex, age, heavy drinking history, smoking, HBsAg, and HCVAb, the ORs (and 95% CIs) for the Ser/Cys, Cys/Cys, and Ser/Cys+Cys/Cys genotypes relative to the Ser/Ser genotype were estimated at 0.79 (0.35-1.79), 0.48 (0.18-1.27), and 0.68 (0.31-1.46) against hospital controls respectively,, and at 1.51 (0.96-3.37), 0.86 (0.50-1.47), and 1.25 (0.82-1.91), against CLD patients respectively.

Table 2 shows the adjusted ORs of HCC for *hOGG1* genotypes according to heavy drinking history and current smoking status. We could not detect any significant linear trend for the genotypes in any stratum. Among those without heavy drinking history, a significant risk excess for the Ser/Cys vs. Ser/Ser genotype (fully-adjusted OR = 1.82, 95% CI: 1.10-3.01) was observed between HCC cases and CLD patients, yet the risk for the Cys/Cys vs. Ser/Ser genotype was not elevated (OR = 0.90, 95% CI: 0.50-1.64). A similar tendency was observed among those without current smoking. In comparison of HCC cases with hospital controls, additional adjustment for HBsAg and HCVAb substantially altered the OR on some occasions (e.g., OR for Cys/Cys among current smokers), but with a very wide CI. No significant interaction was found between the *hOGG1* genotype and either heavy drinking history or current smoking. Although we conducted corresponding analyses based on daily amount of alcohol drinking and pack-years of smoking, the results were essentially identical (data not shown).

**Table 2.** Adjusted odds ratios (ORs) and their 95% confidence intervals (CIs) of HCC for *hOGG1* genotypes according to heavy drinking history and current smoking status.

	HCC cases		Hospital controls	CLD patients	HCC cases vs. hospital controls		HCC cases vs. CLD patients	
					
	n(%)		n(%)	n(%)	OR* (95% CI)	OR^†^ (95% CI)	OR* (95% CI)	OR^†^ (95% CI)
Without heavy alcohol drinking history^‡^								
Ser/Ser	40 (25.0)		64 (25.4)	96 (28.1)	1.00 (reference)	1.00 (reference)	1.00 (reference)	1.00 (reference)
Ser/Cys	88 (55.0)		115 (45.6)	154 (45.0)	1.31 (0.77- 2.22)	0.73 (0.28- 1.93)	1.78 (1.07- 1.63)	1.82 (1.10- 3.01)
Cys/Cys	32 (20.0)		73 (29.0)	92 (26.9)	0.80 (0.43- 1.49)	0.42 (0.13- 1.28)	0.90 (0.50- 1.63)	0.90 (0.50- 1.64)
P for trend					0.51	0.13	0.84	0.86

With heavy alcohol drinking history^‡^								
Ser/Ser	16 (32.7)		9 (39.1)	9 (23.1)	1.00 (reference)	1.00 (reference)	1.00 (reference)	1.00 (reference)
Ser/Cys	22 (44.9)		8 (34.8)	22 (56.4)	1.56 (0.44- 5.59)	0.92 (0.17- 5.06)	0.50 (0.16- 1.50)	0.63 (0.18- 2.19)
Cys/Cys	11 (22.5)		6 (26.1)	8 (20.5)	1.05 (0.24- 4.61)	1.02 (0.10-10.06)	0.70 (0.18- 2.79)	0.84 (0.20- 3.84)
P for trend					0.87	0.99	0.54	0.81
P for interaction^§^					0.94	0.95	0.13	0.16

Without current smoking								
Ser/Ser	35 (25.0)		56 (26.9)	78 (26.4)	1.00 (reference)	1.00 (reference)	1.00 (reference)	1.00 (reference)
Ser/Cys	77 (55.0)		91 (43.8)	136 (46.1)	1.69 (0.95- 3.00)	1.00 (0.38- 2.59)	1.61 (0.95- 2.72)	1.65 (0.97- 2.81)
Cys/Cys	28 (20.0)		61 (29.3)	81 (27.5)	0.83 (0.42- 1.63)	0.60 (0.20- 1.84)	0.82 (0.44- 1.53)	0.86 (0.46-1.61)
P for trend					0.66	0.39	0.61	0.71

With current smoking								
Ser/Ser	21 (30.4)		17 (25.4)	27 (31.4)	1.00 (reference)	1.00 (reference)	1.00 (reference)	1.00 (reference)
Ser/Cys	33 (47.8)		32 (47.8)	40 (46.5)	0.77 (0.31- 1.92)	0.42 (0.07- 2.42)	1.04 (0.43- 2.52)	1.01 (0.41- 2.52)
Cys/Cys	15 (21.7)		18 (26.9)	19 (22.1)	0.81 (0.29- 2.32)	0.07 (0.003- 1.39)	1.00 (0.35- 2.84)	0.92 (0.32- 2.67)
P for trend					0.69	0.08	0.99	0.89
P for interaction^§^					0.15	0.70	0.52	0.69

* : Adjusted for sex, age category (40-49, 50-59, 60-69, and 70-79 years), and either smoking status (never, former, and current smokers) or heavy drinking history.

† : Adjusted for the above factors plus HBsAg and HCVAb.

‡ : A “heavy alcohol drinking history” was defined as having imbibed ≥69 g ethanol per day for ≥10 years.

§ : Calculated by the likelihood ratio test.

HCC : hepatocellular carcinoma

CLD : chronic liver diseases

## DISCUSSION

In the present study, we could not find any significant association between *hOGG1* Ser326Cys polymorphism and overall HCC risk. In subgroup analyses according to drinking and smoking habits, there was some risk increase for the Ser/Cys vs. Ser/Ser genotype, yet such a finding might be due to chance variation in the light of the absence of risk increase for the Cys/Cys vs. Ser/Ser genotype. In addition, no significant gene-alcohol or gene-smoking interaction was evident.

Chronic inflammation caused by hepatotropic viruses and exposure to alcohol and tobacco stimulate hepatic ROS generation,^7^^-^^9^ and some reports also have demonstrated that both HCV and HBV infections could induce ROS without inflammation.^23^^,^^24^ Interestingly, a recent clinical study reported that reducing iron, one of the sources of ROS generation, by phlebotomy and low iron diet decreased hepatic levels of 8-OHdG and eventually the risk of HCC development in patients with chronic hepatitis C after 6 years of follow-up.^25^ These reports suggest an important role of oxidative stress in hepatocarcinogenesis.

hOGG1, which acts in the DNA base excision repair pathway, excises 8-OHdG resulting from oxidative stress. The Ser326Cys polymorphism in *hOGG1* may alter glycosylase function, and some studies showed that hOGG1 protein encoded by the 326Cys had substantially lower DNA repair activity than that encoded by the 326Ser allele in an in vitro Escherichia coli complementation activity assay^14^ and in human cells in *vivo*^15^ whereas others did not find such a difference.^26^^,^^27^ Thus, there is limited evidence of the genotype-phenotype relation, yet recent epidemiologic studies suggested that the putative low active allele (326Cys) was positively associated with lung, orolaryngeal, esophageal, stomach, and colon cancers,^16^^-^^20^ but not with breast cancer.^28^ To our knowledge, this is the first epidemiologic study on the association between the Ser326Cys polymorphism and HCC risk. Despite its high biological plausibility, however, we could not obtain any significant findings.

In this study, selection bias among controls could be responsible for the lack of association. However, we used two different control groups, and the results based on both control groups showed a similar tendency. Furthermore, the observed frequencies of the 326Ser allele (0.49 among hospital controls and 0.51 among CLD patients) were close to those in two earlier case-control studies among the Japanese (0.53 in both).^19^^,^^22^ Given the sample size and the genotype frequency of hospital controls, we had an 83% chance of detecting a doubling of the risk for Ser/Cys+Cys/Cys (putative risk genotypes) vs. Ser/Ser (two-sided p = 0.05).

The difference of hOGG1 activity potentially caused by the Ser326Cys polymorphism may be compensated by higher induction of other cooperative enzymes (e.g., human MTH homolog 1^29^ or human MutY homolog^30^) that prevent 8-OHdG-induced mutagenesis. In addition, regardless of the polymorphism, hOGG1 activity may be functionally inhibited by increased NO production resulting from chronic inflammation, which usually exists as the background of HCC, since NO mediated inhibition of hOGG1 activity has been shown in cholangiocarcinoma cell line.^31^ On the other hand, Sugimura et al^32^ reported that the risk for lung cancer associated with the *hOGG1* Cys/Cys genotype differed by histological subtypes, being elevated for squamous cell carcinoma but not for adenocarcinoma. HCC might represent a histological type unrelated to this genotype.

In conclusion, our results suggest that the *hOGG1* Ser326Cys polymorphism may not play a major role as an independent factor in hepatocarcinogenesis. Although this case-control study of moderate size is among the largest ones that have been reported on the association between HCC and genetic polymorphisms, we could not exclude the possibility of a weak association (e.g., OR < 2.0) with the *hOGG1* polymorphism and its interaction with environmental factors. Further large studies are needed to address these issues.
